# Protocol for quantifying silent ribosome induction in yeast and mammalian cells using polysome profiling

**DOI:** 10.1016/j.xpro.2026.104447

**Published:** 2026-03-20

**Authors:** Sayanur Rahaman, Samuel Mondal, Nicole Schiffelholz, Attila Becskei

**Affiliations:** 1 Biozentrum, Universität Basel, Basel 4056, Switzerland

**Keywords:** Genomics, Molecular Biology, RNA-seq

## Abstract

Translationally silent ribosomes lack bound mRNA and are difficult to quantify. Here, we present a protocol to measure silent ribosome induction under diverse conditions in yeast and mammalian cells. We describe steps to isolate polysome-profiling fractions, analyze ribosome-associated RNA by RNA sequencing with identification and removal of anomalously amplified rRNAs, and validate measurements by qPCR.

For complete details on the use and execution of this protocol, please refer to Rahaman et al.^1^

## Highlights

• Steps for detecting silent ribosomes in eukaryotic cells
• Guidance on polysome profiling and spike-in RNA normalization
• Instructions for correcting anomalous 5S rRNA amplification
• RNA-seq dataset from heat-stressed macrophages

## Abstract (editor highlights)

Publisher’s note: Undertaking any experimental protocol requires adherence to local institutional guidelines for laboratory safety and ethics.

## Graphical abstract

**Figure:**
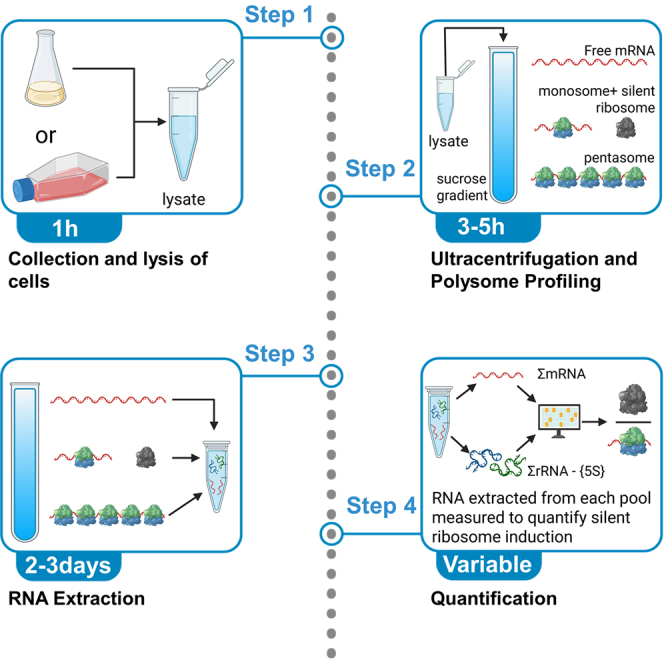

## Before you begin

Translationally silent ribosomes are induced during short- or long-term starvation and under defined stress conditions, representing dormant ribosome states. Although such ribosomes have long been known in prokaryotes, their discovery in eukaryotes lagged behind, partly because they possess few distinctive features detectable by standard analytical methods, most notably the absence of a characteristic disome peak in polysome profiles.^2^

This protocol describes how to obtain accurate mRNA and rRNA measurements from polysome-profiling fractions—using both low- and high-throughput methods—to quantify silent ribosome induction under diverse stress conditions. A central aspect of the procedure is the detection and exclusion of anomalously amplified rRNA. We previously optimized this workflow in yeast and now validate it in mammalian cells, demonstrated here using a macrophage cell line.

The following preparations must be completed before starting the protocol.

1. Prepare the sucrose solutions needed for polysome gradient formation.

***Note:*** Although full recipes are provided in Materials and Equipment Setup, 80% (w/v) sucrose solutions must be prepared in advance so they reach the correct temperature and consistency, by mixing the appropriate amount of pure (>99%) sucrose in nuclease free water. Use of hot water aids dissolution. Complete dissolution of sucrose is essential for generating smooth and reproducible gradients.

2. Grow yeast cultures to mid-log phase.

Yeast must be collected during exponential growth to ensure well-defined polysome profiles.

***Note:*** Mid-log phase cultures are appropriate for the study of short-term starvation and heat-stress.

3. Culture mammalian cells to 70%–80% confluence.

***Note:*** This protocol has been validated in a macrophage cell line; similar confluence levels are suitable for other adherent cell types. Cell density strongly influences translational activity and must be consistent across experimental replicates.

### Innovation

Our protocol introduces methodological improvements in both major stages of the workflow: polysome profiling and RNA quantification.

In the polysome-profiling stage, we address two key challenges. First, because silent ribosomes are induced under starvation or stress, rapid arrest of translation is essential to preserve the in vivo ribosome distribution. We therefore implemented fast cooling procedures that ensure immediate translation shutdown even following heat stress. Second, sample homogeneity must be maintained, as sucrose—the core component of polysome gradients—reduces RNA yield, particularly from polysomal fractions. To improve yield and measurement precision, we dilute pooled polysomal fractions to lower sucrose concentrations and add spike-in control RNA to each fraction before RNA extraction.

In the RNA-quantification stage, our approach provides a quantitative framework to measure ribosomes devoid of mRNA. Specifically, we use the corrected mRNA-to-rRNA ratio as an index for the abundance of silent ribosomes, with lower ratios indicating a higher proportion of ribosomes lacking associated mRNA. Accurate estimation of silent ribosomes requires detection and removal of anomalous rRNA amplification. In our earlier yeast work, we identified substantial overamplification of 5S rRNA in RNA-seq datasets.^1^ Here, we extend this analysis to mammalian cells, specifically macrophages, and observe a similar artifact. Inclusion of all rRNAs leads to a nearly tenfold underestimation of active monosomes under heat stress. After excluding 5S rRNA, the corrected mRNA-to-rRNA ratios reveal that approximately 20% of monosomes remain active in macrophages under these conditions.

### Institutional permissions

Molecular biology activities in our laboratory are registered and overseen by the Biosafety Officer of the Biozentrum, University of Basel, in accordance with institutional biosafety regulations. The experiments described in this protocol did not involve human-derived materials or work requiring additional biosafety authorization and were conducted within this approved framework. Researchers should obtain the appropriate institutional permissions before performing related experiments.

### Preparation of 5× PGB

**Timing: 30 min**

4. Prepare the 5× Polysome Gradient Buffer (PGB) solution as described in the “materials and equipment” section.
5. Filter through a 0.22 mm syringe filter.

**CRITICAL:** This ensures that there is no debris that may impact the gradient.

### Designing primers for qPCR-based quantification

**Timing: Variable, up to 1 week with quality control**

***Optional:*** Only for the qPCR-based quantification.

6. Design forward and reverse qPCR primers against 25S rRNA to yield an amplicon of 75–150 nucleotides.

***Note:*** rRNA has numerous repetitive sequences and it will be necessary to carry out melt curve test to make sure that no primer dimers and spurious products are formed.

7. Identify genes of interest as a set of at least 15 mRNAs that are highly expressed under both standard and stress conditions.
8. Design forward and reverse qPCR primers specific to the mRNAs of interest in such a way that the forward primer contains the start codon on the mRNA to yield an amplicon of 75–150 nucleotides.

### Preparation of sucrose gradients

**Timing: 24 h**

9. Prepare light (e.g., 10% w/v) and heavy (e.g., 50% w/v) solutions of sucrose by mixing 80% (w/v) sucrose with 5x PGB (final concentration 1x) and diluting with an appropriate volume of water and mix for 2–3 h at 4°C with gentle rocking.

***Note:*** The gradients can be adjusted to get a better resolution of particular ribosomal peaks. Make sure to keep the difference between the heavy and light solutions of sucrose to a minimum of 30% to avoid gradient collapse during ultracentrifugation.

10. Degas the solutions by centrifugation at 1500 g for 10 min.
11. Mark the half-way point on the 13.2 mL ultracentrifuge tube with the help of the marker block
12. Pour the light solution till the mark.
13. With a broad-gauge syringe, layer the heavy solution at the bottom of the tube up to the halfway mark. Follow De Siqueira et al.^3^ for a detailed video.
14. Then place the appropriate caps on the tubes and remove excess liquid that may have collected in the cap.
15. Use the Gradient Master part of the Gradient Station Machine to make the gradient. Use the appropriate preset programs corresponding to the gradient used.
16. Keep at 4°C overnight until it is time to load the samples.

**CRITICAL:** Do not introduce bubbles or shake the gradient; even minor disruptions can negatively affect polysome profile resolution.

## Key resources table

**Table:** 

REAGENT or RESOURCE	SOURCE	IDENTIFIER
**Chemicals, peptides, and recombinant proteins**		

Cycloheximide	Thermo Scientific	Cat#357420050
GlycoBlue	Thermo Scientific	Cat#AM9516
RNasIN Plus	Promega	Cat#N2615
SuperScript IV	Thermo Scientific	Cat#18090200
Nuclease Free Water	Thermo Scientific	Cat#AM9932
Random hexamers	Promega	Cat#C1181
oligodT_15_	Promega	Cat#C1101
KAPA SYBR Fast	Sigma Aldrich	Cat#KK4611
Tris-HCl pH 8.0	Thermo Scientific	Cat#AM9856
MgCl_2_	Thermo Scientific	Cat#AM9530G
DTT	Thermo Scientific	Cat#707265mL
KCl	Thermo Scientific	Cat#AM9640G
Cycloheximide (RNA grade)	Thermo Scientific	Cat#J66004-XF
cOmplete EDTA free Protease Inhibitor	Sigma Aldrich	Cat#COEDTAF-RO
Sucrose	Thermo Scientific	Cat#036508-30
Phenol (pH 4.5)	Sigma Aldrich	Cat#P4682-400mL
Chloroform-Isoamyl Alcohol (24:1)	Sigma Aldrich	Cat#25666-500mL
Absolute Ethanol	Sigma Aldrich	Cat#1009831000
Triton X-100	Sigma Aldrich	Cat#T9284
Phosphate buffered saline (PBS)	Sigma Aldrich	Cat#D8537

**Critical commercial assays**		

RNeasy MinElute Cleanup Kit	Qiagen	Cat#74204
RNA Clean and Concentrator	Zymo	Cat#R1013
Qiaseq Fastselect Yeast Kit	Qiagen	Cat#334217
SMART-Seq Stranded	TaKaRa	Cat#634444
TranscriptAid T7 High Yield Transcription Kit	Thermo Scientific	Cat#K0441
TruSeq Stranded kit	Illumina	Cat#20020599

**Deposited data**		

Polysome profiling of murine macrophages without rRNA depletion, at 37° C (Control) and 42° C (Heat-shock)	This paper	GEO: GSE311393

**Experimental models: Cell lines**		

Mouse: J774A.1 Murine macrophages	Laboratory of Prof. Dr. Jean Pieters	ATCC: TIB-67

**Experimental models: Organisms/strains**		

*S. cerevisiae*: Strain background: BY4743	EUROSCARF	Y20000

**Oligonucleotides**		

Sc_RDN25-1_qPCR_ForwardPrimer	AATCTCGCATTTCACTGGGC	9608
Sc_RDN25-1_qPCR_ReversePrimer	TTGACTTACGTCGCAGTCCT	9614
Mm_RN28S1_qPCR_ForwardPrimer	GGGTTTAGACCGTCGTGAGA	10133
Mm_RN28S1_qPCR_ReversePrimer	CTCAGCCAAGCACATACACC	10139

**Other**		

Zirconia/Silica beads	Biospec	Cat#11079105z
Ultracentrifuge tubes 13.2 mL	Beckman Coulter	Cat#C14293
Caps for Ultracentrifuge tubes	BioComp	Cat#105-414-1
SW41-Ti Ultracentrifuge Rotor	Beckman Coulter	Cat#331362
Lysing Matrix Y	MPBio	Cat#116960050-CF
Teenprep™ Lysing Matrix Y tubes	MPBio	Cat#116975050
Lightcycler 480	Roche	RRID:SCR_018626
Gradient Station	BioComp	Cat#153
Marker Block (for SW41-Ti Rotor)	BioComp	Cat#105-614A
23G syringe	B Braun	Cat#4665635
5 mL microfuge tube (DNA Lobind)	Eppendorf	Cat#0030108310s
2 mL microfuge tube (DNA Lobind)	Eppendorf	Cat#0030108078

## Materials and equipment

**Table:** 5x Polysome Gradient Buffer (PGB)

Reagent	Final concentration
Tris-HCl pH 8.0	100 mM
MgCl_2_	50 mM
KCl	250 mM
Dithiothreitol (DTT)	5 mM
Cycloheximide (RNA grade)	1 mg/mL
cOmplete EDTA free Protease Inhibitor	1 tab/10 mL
RNasIN RNase Inhibitor	80 U/mL
Nuclease Free Water	N/A

Store at 4°C for up to 5 days. Alternatively, prepare 5x buffer without DTT, Cycloheximide, protease and RNase inhibitor, which can be stored for room temperature for longer and add these components before each experiment.

***Alternatives:*** KCl can be omitted. In the original PGB, the final concentration at 1× is 50 mM. Increasing the KCl concentration beyond this level may promote dissociation of vacant ribosomes into ribosomal subunits,^2^ which should be avoided. Conversely, reducing the KCl concentration to zero has the advantage of effectively arresting ribosome dynamics, as most translation-dependent reactions cease under these conditions.^4^ In this study, we used 0 mM KCl for yeast samples and 250 mM KCl (5× PGB) for mammalian cells.

**CRITICAL:** Cycloheximide is acutely toxic as it is a eukaryotic translation inhibitor. Be careful not to come in contact with it directly.

**Table:** 1x Polysome Lysis Buffer PLB (PLB)

Reagent	Final concentration
5x PGB	1x
Triton X-100	1 %
Nuclease Free Water	N/A

Store at 4°C for up to 1 day.

**Table:** Yeast cell collection media

Reagent	Final concentration
Cycloheximide	300 μg/mL
Yeast culturing media (e.g., CSM Complete)	N/A

Store at 4°C for up to 1 day.

**Table:** Mammalian cell collection media

Reagent	Final concentration
Cycloheximide	200 μg/mL
1x PBS	N/A

Store at 4°C for up to 1 day.

## Step-by-step method details

### Collection and lysis of cells

**Timing: 1 h**

This section describes the collection of the yeast or mammalian cells and subsequent lysis.

1. Collect the cells as follows:
  a. Option 1: Yeast cells (use at least 2x10^8^ cells):
    i. Add 1x volume of yeast culture (e.g., 30 mL) to 2x volume of Yeast cell collection media (e.g., 60 mL). Immediately mix by inversion and cool in a 50% Ethanol bath at −20°C for 2 mins.
      **CRITICAL:** Use of ice-cold collection media and cooling in a −20°C Ethanol bath helps stop translation process immediately by dropping the temperature in the tube to <10°C, as opposed to stopping it in a gradual manner, which would potentially lead to ribosomal run-off.^5^
    ii. Centrifuge at 1500 g for 10 min in a pre-cooled centrifuge at 4°C.
    iii. Completely remove supernatant by decanting, followed by a brief centrifugation and careful pipetting to remove any remaining liquid and proceed to lysis.
  b. Option 2: Mammalian Cells (use at least 1x10^7^ cells):
    i. Remove media by aspiration and immediately place the culture dish on ice
    ii. Add Mammalian cell collection media cooled to 4°C to the culture dish and scrape the cells.
    iii. Collect the cells in an appropriate tube and centrifuge at 1500 g for 10 min in a pre-cooled centrifuge at 4°C.
    iv. Completely remove supernatant and proceed to lysis.
2. Lyse the cells as follows:
  a. Option 1: Yeast cells:
    i. Re-suspend the cells in 600 μL of 1x PLB on ice.
    ii. Add the mixture to 2 mL microfuge tubes containing 500 μL Zirconia/Silica beads
    iii. Lyse at 4°C in a Thermomixer by intermittent vortexing at 2000 rpm, 30 s on 30 s off, 20 cycles.
  b. Option 2: Mammalian cells:
    i. Re-suspend the cells in 600 μL of 1x PLB on ice.
    ii. Pass the mixture 10 times through a sterile 23G needle.
    iii. Carry out passive lysis by incubating on ice for 10 min.
    iv. Pass the mixture 10 times through a sterile 23G needle.
  c. Centrifuge at 4°C, 10000 g for 10 min and collect the supernatant.
  d. Measure A_260_ using a spectrophotometer.

***Note:*** Due to the presence of RNase Inhibitors, Protease Inhibitors and detergents in PLB, all of which absorb in the UV range, it will be necessary to dilute the sample and the blank (1x PLB) at least 10-fold in nuclease free water for A_260_ measurement.

**Pause point:** Samples may be immediately used for polysome profiling or flash frozen in liquid nitrogen and stored at −80°C for several months. Thaw slowly on ice or a metal block and resuspend before polysome profiling.

### Ultracentrifugation and polysome profiling

**Timing: 3**–**5 h**

This section describes the ultracentrifugation of aforementioned cell lysate on a sucrose gradient, followed by polysome profile.

3. Pre-cool the ultracentrifuge to 4°C.
4. Layer 2–5 A_260_ units of the lysate on the top of the sucrose gradients in the ultracentrifuge tube.

**CRITICAL:** Be careful to layer slowly and not disrupt the gradient.

***Note:*** Too little sample added can lead to less defined peaks, as can overloading the tube with too much sample (troubleshooting 1).

5. Weigh the ultracentrifuge tubes along with the buckets and make sure they are balanced.

***Note:*** Here due to the high forces during ultracentrifugation, it is necessary that the tubes are balanced to a maximum difference of 10 mg in weight.

6. Load the buckets containing the tubes into the rotor and carry out the ultracentrifugation at 275000 g (equivalent to 40,000 rpm in an SW41Ti rotor) for 2 h.

***Note:*** The ultracentrifugation speed and time can be adjusted to get a better resolution of particular ribosomal peaks (troubleshooting 2).

7. When the ultracentrifugation is about to finish (<10 min left), switch on the Gradient Station machine and carry out the cleaning, blanking and general quality control steps as instructed in the TRIAX program in the computer attached to the Gradient Station.
8. Load 2 mL tubes in the fractionation rack (code 19) as instructed and place it in the Gilson collector unit.
9. Carefully take out the rotor from the ultracentrifuge and keep the buckets with the tubes at 4°C without disturbing the gradients.
10. Carefully take out the ultracentrifuge tube from the bucket and place it in the tube holder from the Gradient station machine under the piston.
11. Fractionate the gradient, collect the fractionated samples and save the polysome profile trace generated by the TRIAX program.
12. Keep the fractions in ice if used immediately or store at −80°C.

***Note:*** For a detailed account of using the Gradient Station machine, follow the manufacturer’s instructions or refer to De Siqueira et al.^3^

**Pause point:** The fractions can be stored at −80°C for several months.

### RNA extraction

**Timing: 2**–**3 days**

This section describes how the polysome profile fractions are pooled and how RNA is recovered and purified from them.

**Figure 1:**
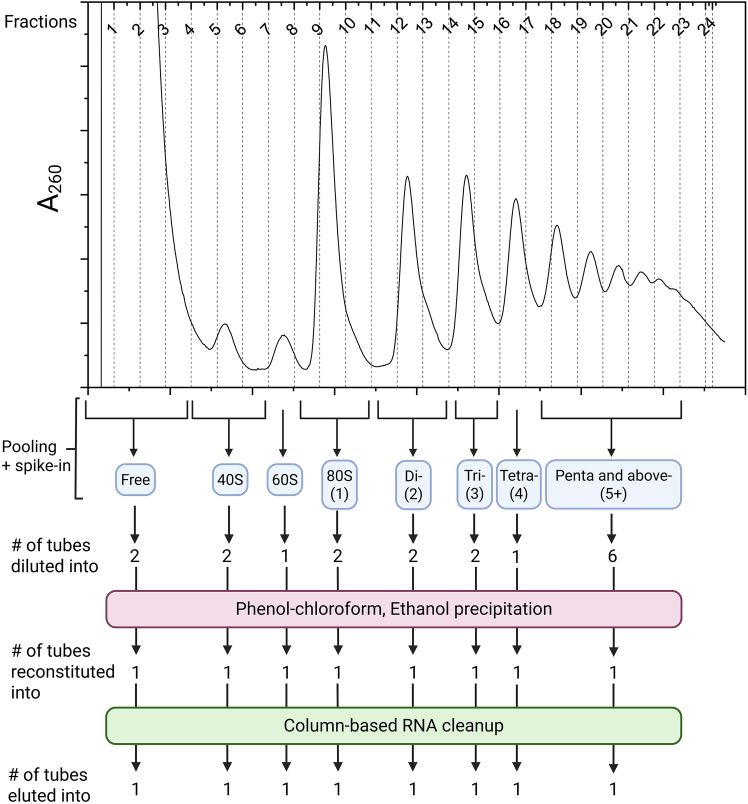
Scheme for RNA extraction from polysome profile fractions The fractions generated from the polysome profile were first pooled as shown and a unit volume of spike-in RNAs was added to each pool. The pools were then diluted into multiple tubes using the considerations explained in steps 15–17. Following this, the RNA was cleaned up by phenol-chloroform and ethanol precipitation and then the diluted tubes from each pool were merged. Finally, a second, column-based cleanup was carried out for each pool.

General outline is given in Figure 1.

13. Prepare Phenol:Chloroform:Isoamyl Alcohol = 25:24:1 mix by mixing equal volumes of acid phenol (pH = 4.5) and chloroform:isoamyl alcohol (24:1) and keep at 37°C.
14. From the polysome profile trace, identify the fractions (collected in step 11) corresponding to a particular mRNA-ribosome complexation state (e. Free, 40S, 60S, Monosome (80S), Disome, etc.)
15. Pool the fractions corresponding to each particular mRNA-ribosome complexation state together in a 5 mL microfuge tube and add equal volumes of spike-in RNAs to each pool.

***Note:*** The spike ins can be prepared by *in-vitro* translation (IVT) or may be purchased as ready to use mixes (e.g. ERCC).

**CRITICAL:** The amount of spike-in RNAs to be added needs to be standardized in a prior experiment, to ensure that they are in the detectable range while measuring the final readout with RT-qPCR or RNA-seq.

16. If any pool exceeds 1200 μL, divide it into multiple 5-mL microfuge tubes as needed. Add nuclease free water to a final volume of 1200 μL.
17. If any tube contains a pooled sample with a sucrose concentration >30%, split into multiple 5 mL microfuge tubes as needed. Add nuclease free water to a final volume of 1200 μL.

***Note:*** Here the concentration of sucrose in the tube can be inferred from the location of the corresponding peak in the polysome profile assuming a linear gradient of sucrose and taking into account the dilution due to water addition in the tube. Too high sucrose concentration can reduce RNA yield (troubleshooting 3).

18. Add 1200 μL of the phenol-chloroform-isoamyl alcohol mix (prepared in step 13, kept at 37°C) to each 5 mL microfuge tube containing 1200 μL of sample. Mix by vortexing and let stand for 1 min.

***Note:*** In this step, the effect of temperature in ensuring the separation of RNA into the aqueous phase outweighs the potential damage to RNA.

19. Centrifuge the 5 mL microfuge tubes at 40°C for 10 min at a speed of 16000 g.
20. Pipet out 1 mL of the upper (aqueous) phase into a fresh 5 mL microfuge tube.
21. Add 100 μL of Sodium acetate (pH = 5.0), 2 μL of glycoblue and 3.3 mL of absolute ethanol.
22. Vortex the tubes and keep at −80°C overnight for precipitation.

**Pause point:** The tubes can be stored at −80°C for up to a year.

23. Prepare 75% ethanol and keep chilled in ice.
24. Centrifuge the tubes at 4°C for 1 h at a speed of 20000 g.
25. Discard the supernatant while taking care to not disturb the pellet (blue).
26. Add 2 mL of 75% ethanol and vortex to wash the pellet.
27. Centrifuge at 4°C for 10 min at a speed of 20000 g.
28. Completely remove any traces of ethanol and let dry for 1 min on the bench.
29. Reconstitute the pellets from each pool in warm nuclease free water (at 50°C). For pools which have been separated into multiple tubes, dissolve one pellet first and then use the resulting solution to dissolve the next one from the same pool.

***Note:*** At this point, the RNA from each pool corresponding to a particular mRNA-ribosome complexation state is reconstituted in 100 μL of nuclease free water.

**Pause point:** The RNA can be stored at −80°C for several months.

30. Further purify this RNA using column-based cleanup (e.g., RNeasy Minelute) including the on-column DNase digestion step. Elute in 30 μL of nuclease free water.

**CRITICAL:** The use of the on-column DNase digestion ensures the removal of DNA which is a common contaminant.

***Alternatives:*** RNeasy MinElute kit contains columns that select for RNA >200 nucleotides. This is helpful to deplete 5S rRNA which can lead to spurious reads in the RNA-seq as well as anomalous amplification of 5S rRNA in the qPCR.^1^ If RNA species with lengths <200 nt are to be studied, Zymo RNA Clean and Concentrator can be used.

### Quantification

**Timing: 2 days for qPCR option, variable for RNA-seq option**

This section describes how the induction of silent ribosomes is quantified using the RNA recovered from each pooled fraction.

**Figure 2:**
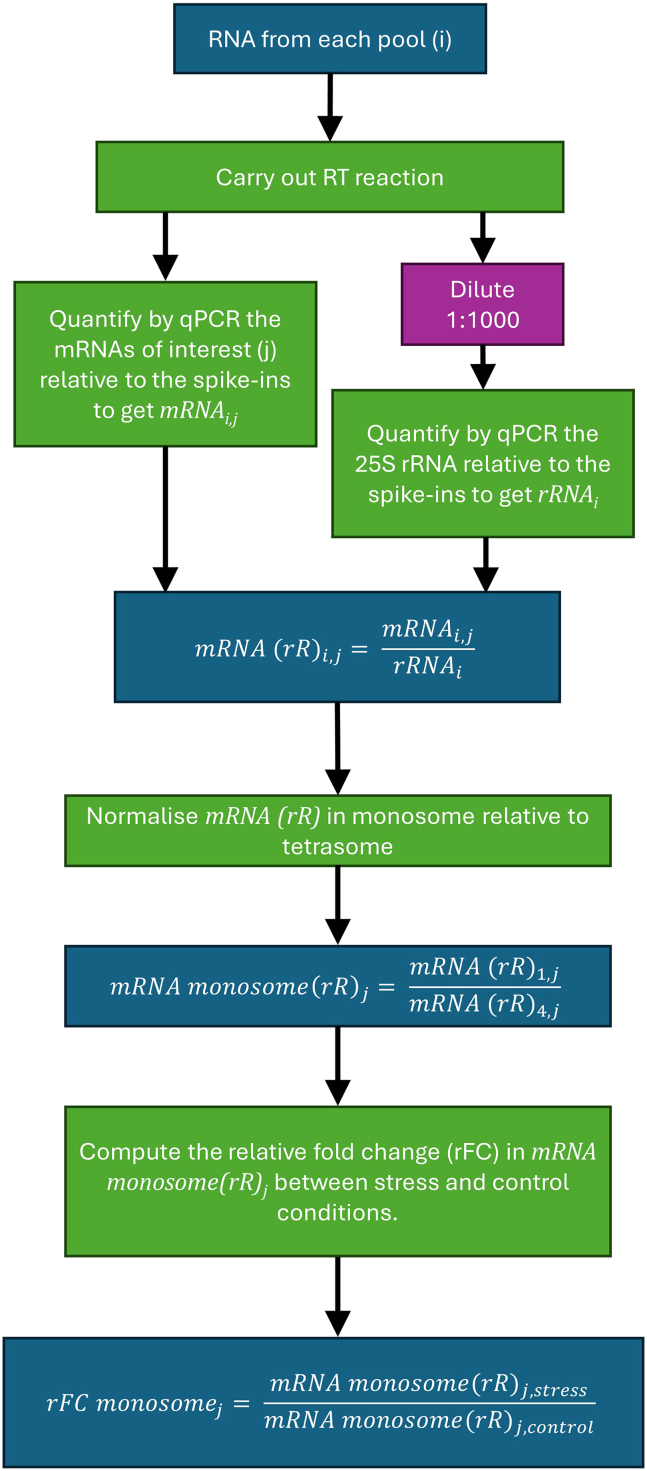
Scheme for quantifying silent ribosome induction by qPCR Scheme shows an outline of the processes detailed in step 31.a.
  b. Option 2: RNA-Seq: Scheme in Figure 3.
    i. Quantify the eluted RNA from step 30 using Qubit or any other fluorescence-based measurement technique. troubleshooting 5.
    ii. Carry out library preparation on the RNA using SMART-Seq Stranded kit if using yeast samples and TruSeq Stranded kit if using mammalian samples. Follow the kit instructions for library preparation and downstream quality control.
      **CRITICAL:** Since here we are using the rRNA levels as a readout for ribosomes and using this to calculate silent ribosomes, it is imperative that no mRNA enrichment or rRNA depletion step is carried out.
    iii. Carry out RNA-Seq on the prepared libraries.
    iv. Trim the reads from the RNA-Seq using TrimGalore and map them onto the respective transcriptome (along with the spike-ins) using Salmon.^6^
    v. Express the reads as transcripts-per-million (TPM) and normalize them to the spike-in values.
    vi. For each pool, calculate mRNA relative to rRNA as follows:
      $$mRNA\left(rR\right)=\frac{\sum mRNA}{\sum \left(rRNA-\left\{5S\right\}\right)}$$
      ***Note:*** 5S rRNA has been found to display anomalous amplification which may be linked to its small size. As a result, it is not used as a readout for rRNA levels.^1^
    vii. Normalize the ratio of mRNA to rRNA in monosome (1) relative to tetrasome (4) as follows:
      $$mRNA\;monosome\left(rR\right)=\frac{{mRNA\left(rR\right)}_{1}}{{mRNA\left(rR\right)}_{4}}$$
      ***Note:*** To estimate the fold change in active monosomes, the mRNA-to-rRNA ratio must be normalized to a stable polysomal reference. In principle, any polysomal fraction containing two or more ribosomes could serve as such a reference. However, this choice involves opposing constraints. Polysomal fractions with high ribosome numbers typically contain mRNAs of low abundance, resulting in increased noise. Conversely, disomes are also suboptimal as a reference for two reasons: they may partially overlap with the monosome peak, and, in some eukaryotes, disomes can occasionally be vacant.^2^ Tetrasomes therefore provide a suitable compromise, as they are well separated from monosomes, sufficiently abundant, and reliably represent actively translating ribosomes.
    viii. Compute the relative fold change (rFC) in monosomal mRNA/rRNA ratios between stress (e.g., heat shock) and control conditions:

31. Measure the RNA intensities as follows:
  a. Option 1: qPCR: Scheme in Figure 2.
    i. Set up a reverse transcription reaction with 5 μL of the eluted RNA using SuperScript IV. Use oligo(dT)_15_, 25S rRNA reverse primer as well as the reverse primers for the spike-in RNAs as the RT primers.
    ii. Carry out reverse transcription at 50°C for 1 h.
    iii. Probe the cDNA generated in step i. for the genes of interest by qPCR using primers flanking the start codon as described in preparation step “designing primers for qPCR-based quantification,” while also measuring the spike-in RNAs as control.
    iv. Dilute the cDNA generated in step i. 1:1000 and probe for 25S rRNA by qPCR, while also measuring the spike-in RNAs as control.
      ***Note:*** 25S rRNA (present in the large ribosomal subunit) rather than the 18S rRNA (present in the small subunit) was used in order to prevent signal from the scanning 40S ribosomal subunits that could be associated with mRNAs in each peak of the polysome profile (troubleshooting 4).
      **CRITICAL:** If this heavy dilution is not carried out, the rRNA Ct values will be too low to be measured correctly.
    v. Normalize both the mRNAs of interest (j) and the 25S rRNA to the spike-in RNA intensities for each pool (i) to get mRNA_i,j_ and rRNAj respectively.
    vi. Calculate mRNA relative to rRNA for each mRNA of interest in each pool as follows:
      $${mRNA\left(rR\right)}_{i,j}=\frac{{mRNA}_{i,j}}{{rRNA}_{i}}$$
    vii. For each mRNA, normalize the ratio of mRNA to rRNA in monosome relative to tetrasome, as follows:
      $${mRNA\;Mono/Tetra\;\left(rR\right)}_{j}=\frac{{mRNA\left(rR\right)}_{1,j}}{{mRNA\left(rR\right)}_{4,j}}$$
      ***Note:*** Here *1* refers to the monosome, *4* to the tetrasome (4 ribosomes) and *j* is the species of mRNA.
    viii. Compute the relative fold change (rFC) in monosomal mRNA/rRNA ratios between stress (e.g., heat shock) and control conditions for each mRNA species *j*:
      $${rFC\;monosome}_{j}=\frac{{mRNA\;Mono/Tetra\left(rR\right)}_{j,stress}}{{mRNA\;Mono/Tetra\left(rR\right)}_{j,control}}$$
      ***Note:*** A lower rFC monosome value indicates a higher prevalence of silent ribosomes in stress condition.

**Figure 3:**
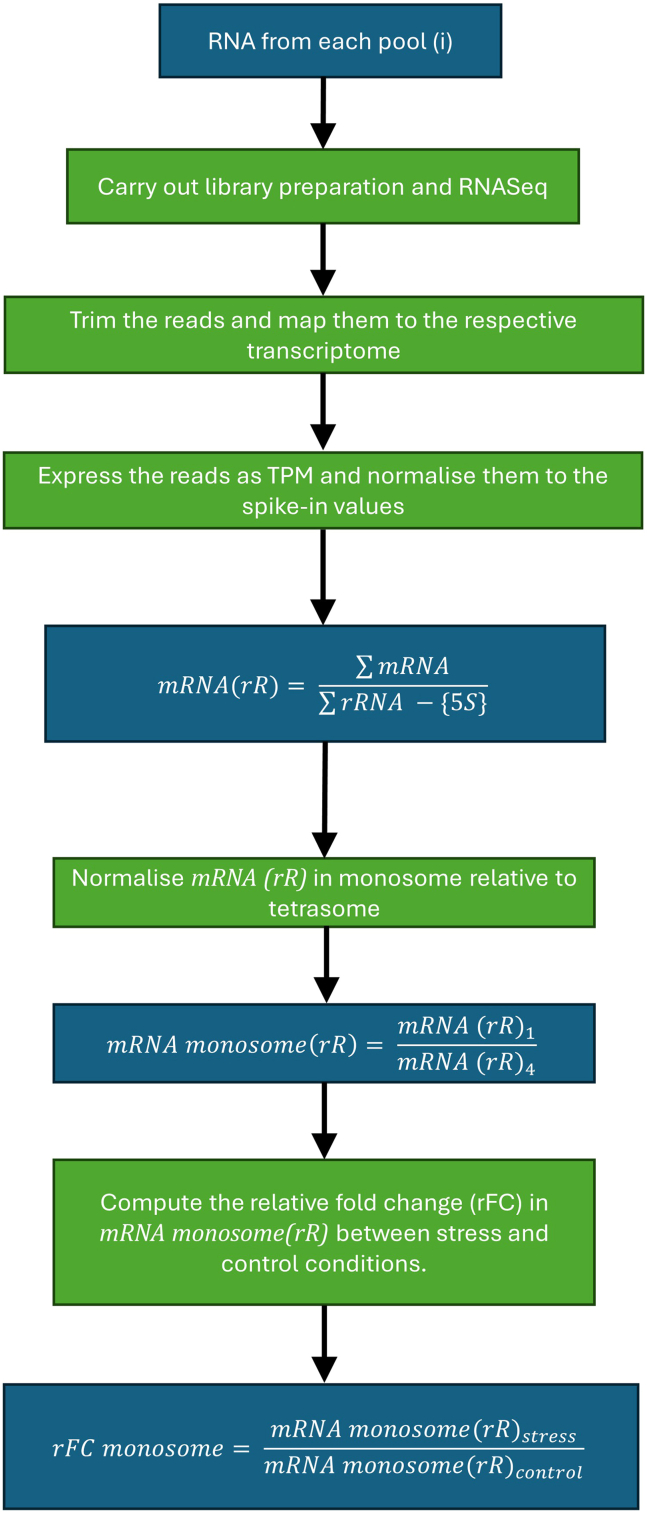
Scheme for quantifying silent ribosome induction by RNA-seq Scheme shows an outline of the processes detailed in step 31.b.

$$rFC\;monosome=\frac{{mRNA\;monosome\left(rR\right)}_{stress}}{{mRNA\;monosome\;\left(rR\right)}_{control}}$$

## Expected outcomes

### Polysome profile quality and RNA yield

Successful polysome profiling should yield a trace with well-defined and well-separated peaks (Figure 1). Under optimal conditions in yeast, peaks corresponding to mRNAs associated with up to 9-10 ribosomes can typically be resolved. Adding cycloheximide 2 min before harvesting prevents ribosome run-off and stabilizes polysomes. In addition, rapid cooling is required to arrest translation. This step becomes more challenging after heat shock, as high-temperature medium must be cooled immediately; this can be achieved by placing the culture – collection media mix in a 50% ethanol bath at −20°C for 2 mins (See step 1.a.i).

**Figure 4:**
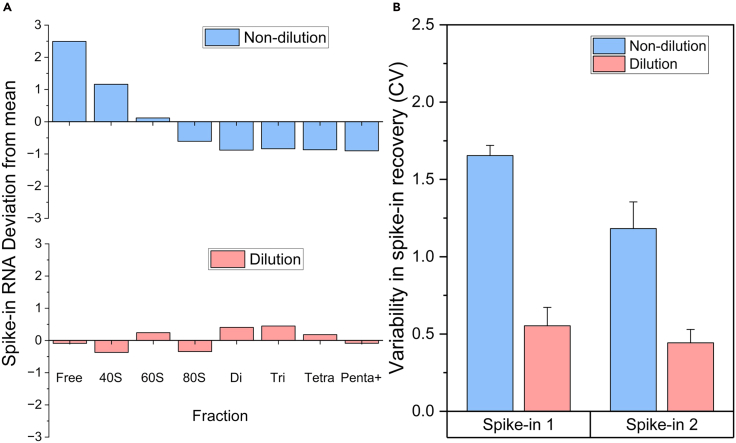
Dilution of the polysome profiling fractions leads to consistent RNA extraction (A) Comparison of two similar polysome profiling experiments which differed only in the fact that in one, the fractions were pooled and diluted as described in this paper and in the other, they were extracted without dilution. We see that in the non-diluted sample there is a greater variation in the spike-in intensities and especially the heavier polysomes which would have higher sucrose yield lesser RNA. (B) The mean CV of the spike-in RNAs across all pools/fractions, calculated for two different spike-ins, for the experiments in which the fractions were diluted (red) and non-diluted (blue). Error bars show standard error of the mean (SEM), N=2 replicates.

RNA yields from individual pooled fractions corresponding to one or more ribosome peaks should exceed 30 ng as measured by Qubit. Spike-in RNA intensities should display low variability (CV < 1.0) across fractions. Variability can be reduced by diluting RNA precipitates to lower the effective sucrose concentration, which otherwise compromises RNA recovery from sucrose-rich fractions (Figure 4). Such dilution is not required if each fraction is precipitated, purified, and sequenced individually,^7^ although this approach is more cost-intensive. When fractions are not pooled, the amount of the sucrose in individual 1.5 mL tubes is less and thus precipitation is likely less inhibited.

For calculating silent ribosome induction, the critical fractions are those representing the monosome and the polysomal fractions containing four ribosomes. If the four-ribosome (tetrasome) fraction does not provide sufficient RNA, higher polysomal fractions (up to 7–10 ribosomes) should be included in the pool.

### Comparison of mRNA distribution and absorbance profiles

As a first step, the mRNA distribution across fractions is measured to characterize the translation apparatus. Several additional parameters can be assessed to evaluate ribosome distribution, including protein abundance of ribosomal and associated factors (proteomics), rRNA quantification by qPCR or RNA-seq,^1^^,^^8^ and the A_260_ absorbance profile, which reflects total RNA and proteins in and associated with the ribosome.

**Figure 5:**
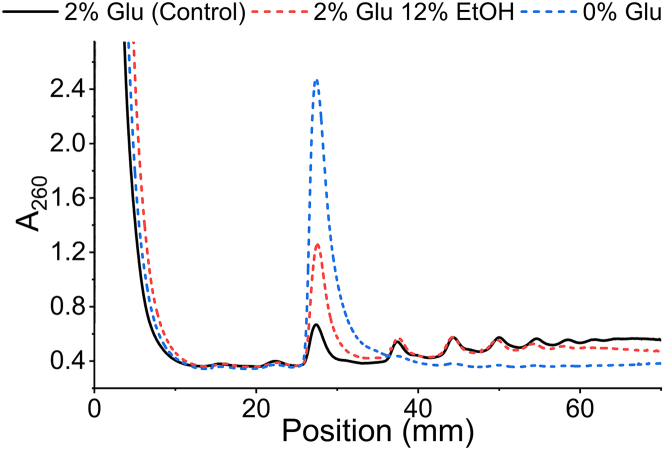
Polysome profile under standard and stress conditions Polysome profile traces of BY4743 cells under standard conditions (2% glucose, black solid line), ethanol stress (12% EtOH, 20 min, red dashed line), and glucose depletion stress (0% glucose, 20 min, blue dashed line).

Because absorbance peaks are inherently obtained during polysome profiling, it is convenient to analyze these first to determine whether the expected patterns are present. In both yeast and macrophages, stress conditions typically produce an increase in the monosome absorbance peak accompanied by a reduction in polysomal peaks (Figure 5).

However, the mRNA distribution does not always mirror these changes. In yeast, mRNAs do not shift toward monosomes—whether quantified by monosome:polysome ratios or polysome propensity.^1^ Thus, monosome accumulation is not accompanied by increased monosome-associated mRNA, representing the first evidence for silent ribosome induction.

### Detection and correction of anomalous 5S rRNA amplification

**Figure 6:**
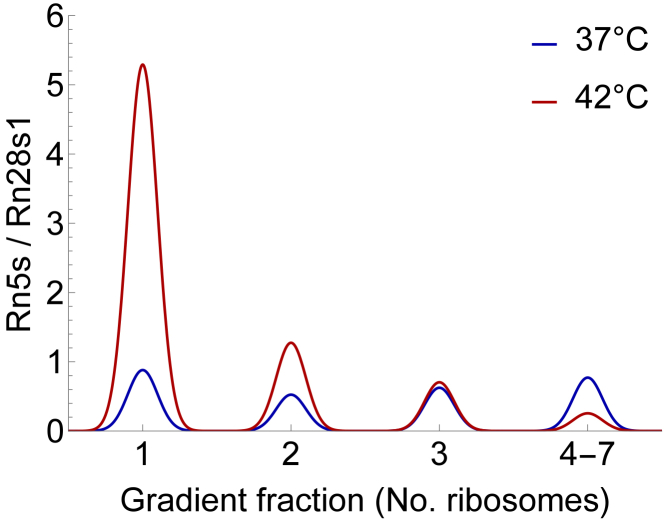
Consistency of the 5S/28S (Rn5S/Rn28S1) rRNA ratios measured with RNA-seq in macrophages No additional column-based purification was performed to remove 5S rRNA. The non-depleted RNA samples isolated from the polysome profiling fractions are shown at the indicated ribosome numbers. The peak values of the bell-shaped curves denote the ratio. Bell-shaped curves are schematic and serve only as positional markers.

In the second step, mRNAs and rRNAs are quantified by RNA-seq. Because rRNA is prone to anomalous amplification, it must be examined first. In our previous yeast work, 5S rRNA appeared strongly overrepresented under heat stress, although this increase was artefactual as confirmed by gel electrophoresis.^1^ Our macrophage data demonstrate that this artifact is conserved in mammalian cells (Figure 6). The mouse genome encodes numerous 5S rRNA variants (Rn5S, n-R5s2 to n-R5s195), which together constitute 80–90% of total RNA TPMs (Data S1).

The Rn5S:Rn28S1 ratio typically remains stable across fractions at 37 °C but increases sharply in monosomes (and more modestly in disomes) at 42 °C, which suggests that anomalous amplification is specific to the stress conditions (Figure 6). This parallels our yeast findings and confirms that the extreme enrichment of 5S rRNA is spurious. Anomalous amplification can be verified by quantifying rRNAs using gel electrophoresis.^1^ Therefore, all 5S rRNA species are excluded from total rRNA calculations (see Step 31.b.vi). After this correction, the ∑*mRNA*/∑(*rRNA*- {5*S*}) ratio is calculated for each fraction.

### Quantitative estimation of active vs. silent monosomes

**Figure 7:**
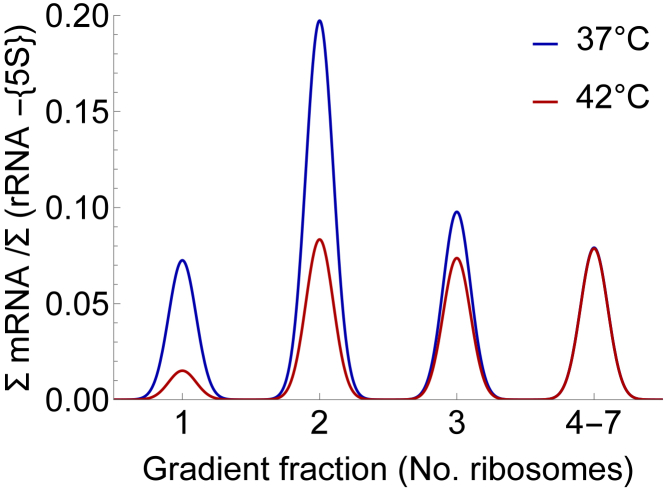
Estimation of the silent ribosomes using RNA-seq in macrophages RNA-seq data obtained from RNA samples in which rRNA was not depleted. mRNAs and rRNAs (5.8S, 18S, and 25S) levels were summed in TPM units to calculate the ∑mRNA/∑(rRNA-{5S}) ratio. The peak values of the bell-shaped curves denote the ratios at 37°C (control) and 42°C (heat stress). Bell-shaped curves are schematic and serve only as positional markers.

Typically, stress reduces the ∑*mRNA*/∑(*rRNA*- {5*S*}) ratio in the monosome fraction (Figure 7). To estimate the fold change in active monosomes, the above ratio must be compared with that of a stable polysomal reference. Tetrasomes represent a suitable reference, as the ∑*mRNA*/∑(*rRNA*- {5*S*}) ratios are nearly identical under normal and stress conditions, and this identity is observed within each system, in both yeast and macrophages (Figure 7).^1^ This normalization effectively accounts for global changes in mRNA transcription and degradation, which would otherwise affect total mRNA levels. When the tetrasome ratios remain unchanged, the fold change in silent ribosomes (see Step 31.b.viii) simplifies and is determined solely by changes in the monosome ratios.

Applying this approach, an estimated 20% of the monosomes remain active in macrophages after heat stress (i.e., 80% become vacant). This estimate is similar to that obtained in yeast under heat stress.

In yeast, qPCR was also used as a rapid alternative to RNA-seq for estimating silent ribosome levels under additional conditions. Accurate measurement using the qPCR option requires careful selection of a set of mRNAs (at least ten) that are highly expressed under both standard and stress conditions. Using such a set, we obtained a narrow spread of silent ribosome estimates. This both validated the RNA-seq results and, owing to the stable mean value, enabled the use of qPCR as a faster alternative for estimating silent ribosome induction under other stress conditions.^1^ A larger gene set will likely be needed for mammalian cells, as silent ribosome induction may coincide with an elongation block (see next section). Under these conditions, a broader spread of values is expected, because some mRNAs may stall early during elongation and accumulate in the monosome fraction, whereas others do not. If the number of mRNAs required becomes too large, RNA-seq represents the more economical and robust alternative.

### Integrating estimation of silent ribosomes with other parameters

In macrophages, mRNAs shift somewhat toward monosomes under heat stress.^1^ This raises the possibility of an additional regulatory layer often attributed to stress-induced slowing or blocking of elongation, or to ribosomal collisions,^9^ thereby making ribosome-position analyses particularly informative. Several studies indeed report ribosome accumulation near the start codon, consistent with altered elongation dynamics.^10^^,^^11^ Ribosome footprinting can reveal such features because, unlike polysome profiling, it uses RNase to digest unprotected regions of mRNA, thereby mapping ribosome positions and indicating potential elongation blocks. However, when both positional information and estimates of silent ribosome formation are required, a stoichiometric analysis becomes necessary. A method based on stoichiometric mRNA:rRNA ratios after digestion has been used to estimate silent ribosome levels under normal conditions.^12^ The results in mammalian cells presented here support the use of such analyses under standard growth conditions, but stress-dependent adjustments may be required to develop a method that takes into account possible rRNA amplification. It is important to note that RNase I digests not only mRNA but also parts of rRNA, potentially altering the degree of anomalous rRNA amplification.

Finally, silent ribosome induction can be complemented by proteomic analyses,^1^ which may reveal additional signatures of translational dormancy.

## Limitations

This protocol measures the fold change in silent ribosomes across conditions; it does not provide the absolute number or percentage of silent ribosomes under any specific condition.

Polysome profile traces may vary across cell types or experimental conditions. In such cases, standardization of ultracentrifugation parameters—including run speed, run time, and gradient composition—may be required.

## Troubleshooting

### Problem 1

Peaks are not well defined.

### Potential solution

This may be due to loading too little sample. If too little sample is loaded, there are essentially a low amount of RNA and ribosomes that are ultracentrifuged. Since the gradient solution itself absorbs at 260 nm, too little sample may lead to the absorbance of some or all peaks to not be enough to be noticeably above the background.

Another (albeit counterintuitive) reason might be due to adding too much sample. Too much sample if added will not pass through the sucrose gradient during the ultracentrifugation run and will thus lead to peaks with low resolution.

We found that using an amount of sample corresponding to 2-5 A_260_ units leads to a polysome profile with adequate resolution.

### Problem 2

Peaks are too congested for fractionation.

The polysome profile is dependent among other things, on the type of cells used and the conditions. For a particular gradient and a particular speed as well as runtime of centrifugation, different cells will give different polysome profile traces as will different conditions for the same type of cells. Under these circumstances, some fractions might encompass more than one peak or one peak might get split across two fractions.

### Potential solution

This will require some degree of standardization. The speed of the ultracentrifugation can be varied as can its runtime. The gradient can also be changed.

A higher speed and/or runtime of the ultracentrifugation will push the contents of the cell lysate further up the gradient (i.e., towards the bottom of the tube). This will have the effect of essentially pushing the gradient towards the right.

Changing the gradient will have a different effect. Increasing the sucrose % in both the light and heavy sucrose solutions will lead to the lighter peaks (free, 40S, 60S, etc) to be compressed while the heavier peaks will have greater resolution, while decreasing the sucrose % will have the opposite effect. Decreasing the difference in sucrose % between the light and heavy solutions will lead to the peaks in the middle of the polysome profile (80S, disome, trisome) to be more compressed.

***Note:*** If the gradient is changed, a different program will need to be used in the gradient station machine to prepare the gradients. The gradient station machine has numerous different preset gradients that the user can make.

### Problem 3

Spike in levels show a large deviation across pools.

Since equal quantities of the spike-in RNAs were added to each pool, they should have equal intensities at the end. Them having a large deviation (we used the cutoff of CV > 1.0) is indicative of RNA loss.

### Potential solution

This loss in RNA generally happens due to sucrose which can inhibit the pelleting of RNA in the ethanol precipitation. Generally, the fractions with higher sucrose concentration show higher loss (Figure 4). The solution to this is to dilute the pools as described in steps 31–33. If the final sucrose concentration is below 25%, it should not have a negative effect on the RNA extraction.

### Problem 4

Low Ct value of 25S rRNA

rRNA constitutes more than 95% of the transcriptome. Being a structural part of the large subunit of the ribosome, 25S rRNA is highly expressed in the cell which can lead to low Ct values (i.e., very early amplification that compromises precision) in qRT-PCR. In addition, rRNA has highly repetitive sequences which might lead to spurious amplification due to self-priming during qRT-PCR, leading to low Ct values.

### Potential solution

We recommend at least a 1:1000 dilution of the cDNA resulting from the RT reaction to get Ct values for 25S rRNA in the quantifiable range. Depending on the resulting Ct values, the cDNA might need to be diluted further. Primers for 25S rRNA will also need to be carefully designed and validated through primer efficiency measurements to ensure there is no self-priming.

### Problem 5

Low RNA yield.

This can happen due to either using too little starting sample (we recommend the sample to be equivalent to 2–5 A_260_ units), loss of sample during RNA extraction, or degradation of RNA.

### Potential solution

Gradients, samples, RNA must be handled in a sterile manner with RNase free reagents and at 4°C, unless otherwise stated.

## Resource availability

### Lead contact

Further information and requests for resources and reagents should be directed to and will be fulfilled by the lead contact, Attila Becskei (attila.becskei@unibas.ch).

### Technical contact

Technical questions on executing this protocol should be directed to and will be answered by the technical contact, Sayanur Rahaman (sayanur.rahaman@unibas.ch).

### Materials availability

This protocol did not generate any new unique reagents.

### Data and code availability

RNA-seq data for polysome profiling of J774A.1 macrophage samples at 37°C and 42°C without rRNA depletion have been deposited in the GEO database of the NCBI (GEO: GSE311393).

## Acknowledgments

We thank Phillippe Demougin (Genomics Facility, University of Basel/BSSE) for the preparation of Illumina libraries and Nitish Mittal for experimental assistance. J774A.1 murine macrophage cells were provided by Jean Pieters. This work was supported in part by the 10.13039/501100001711Swiss National Science Foundation (310030_185001).

## Author contributions

S.R. and A.B. designed the experiments. S.R. and N.S. carried out the experiments. S.R., A.B., and S.M. analyzed the data. S.R. and A.B. wrote the manuscript.

## Declaration of interests

The authors declare no competing interests.

## Declaration of generative AI and AI-assisted technologies in the writing process

During the preparation of this work, the authors used ChatGPT for text corrections. After using this tool, the authors reviewed and edited the content as needed.

## Supplemental information

Data S1 RNA-seq–derived mRNA and rRNA abundance values (TPM) from polysome profiling fractions of macrophages under control and heat-shock conditions, related to Step 31.b. (A) mRNAs (in TPM). (B) rRNAs (in TPM) and calculation of silent ribosome induction. (C) Keys to sheets A and B.

## References

[1] Rahaman S., Schiffelholz N., Mittal N., Fröhlich K.E., Zavolan M., Becskei A. (2025). Heat shock induces silent ribosomes and reorganizes mRNA turnover. Cell Rep..

[2] Delaney C.E., Becskei A. (2025). Detection and Characterization of the Eukaryotic Vacant Ribosome. Int. J. Mol. Sci..

[3] De Siqueira M.K., Nouhi Z., Zhao Y., Wang S., Xiao X., Yang X., Hulea L., Villanueva C.J. (2025). Protocol to perform polysome profiling in primary differentiating murine adipocytes. STAR Protoc..

[4] Blobel G., Sabatini D. (1971). Dissociation of mammalian polyribosomes into subunits by puromycin. Proc. Natl. Acad. Sci. USA.

[5] Mašek T., Valášek L., Pospíšek M. (2011). Polysome analysis and RNA purification from sucrose gradients. Methods Mol. Biol..

[6] Patro R., Duggal G., Love M.I., Irizarry R.A., Kingsford C. (2017). Salmon provides fast and bias-aware quantification of transcript expression. Nat. Methods.

[7] Rahaman S., Faravelli S., Voegeli S., Becskei A. (2023). Polysome propensity and tunable thresholds in coding sequence length enable differential mRNA stability. Sci. Adv..

[8] Jaquet V., Wallerich S., Voegeli S., Túrós D., Viloria E.C., Becskei A. (2022). Determinants of the temperature adaptation of mRNA degradation. Nucleic Acids Res..

[9] Huso V.L., Niu S., Catipovic M.A., Saba J.A., Denk T., Park E., Cheng J., Berninghausen O., Becker T., Green R., Beckmann R. (2026). ZAK activation at the collided ribosome. Nature.

[10] Shalgi R., Hurt J.A., Krykbaeva I., Taipale M., Lindquist S., Burge C.B. (2013). Widespread Regulation of Translation by Elongation Pausing in Heat Shock. Mol. Cell.

[11] Liu B., Han Y., Qian S.B. (2013). Cotranslational response to proteotoxic stress by elongation pausing of ribosomes. Mol. Cell.

[12] Liu B., Qian S.B. (2016). Characterizing inactive ribosomes in translational profiling. Translation (Austin).

